# Metabolite Profile of Cervicovaginal Fluids from Early Pregnancy Is Not Predictive of Spontaneous Preterm Birth

**DOI:** 10.3390/ijms161126052

**Published:** 2015-11-19

**Authors:** Melinda M. Thomas, Karolina Sulek, Elizabeth J. McKenzie, Beatrix Jones, Ting-Li Han, Silas G. Villas-Boas, Louise C. Kenny, Lesley M. E. McCowan, Philip N. Baker

**Affiliations:** 1 Liggins Institute, University of Auckland, 85 Park Road, Auckland 1023, New Zealand; m.thomas@auckland.ac.nz (M.M.T.); k.sulek@auckland.ac.nz (K.S.); t.han@auckland.ac.nz (T.-L.H.); philip.baker@auckland.ac.nz (P.N.B.); 2 Institute of Natural and Mathematical Sciences, Massey University, Albany Campus, Auckland 0632, New Zealand; m.b.jones@massey.ac.nz; 3 School of Biological Sciences, University of Auckland, 3a Symonds Street, Auckland 1010, New Zealand; s.villas-boas@auckland.ac.nz; 4 The Irish Centre for Fetal and Neonatal Translational Research, University College Cork, Wilton 06897, Cork, Ireland; l.kenny@ucc.ie; 5 Department of Obstetrics and Gynaecology, University of Auckland, 2 Park Road, Auckland 1023, New Zealand; l.mccowan@auckland.ac.nz

**Keywords:** metabolomics, cervicovaginal, biomarkers, spontaneous preterm birth

## Abstract

In our study, we used a mass spectrometry-based metabolomic approach to search for biomarkers that may act as early indicators of spontaneous preterm birth (sPTB). Samples were selected as a nested case-control study from the Screening for Pregnancy Endpoints (SCOPE) biobank in Auckland, New Zealand. Cervicovaginal swabs were collected at 20 weeks from women who were originally assessed as being at low risk of sPTB. Samples were analysed using gas chromatography-mass spectrometry (GC-MS). Despite the low amount of biomass (16–23 mg), 112 compounds were detected. Statistical analysis showed no significant correlations with sPTB. Comparison of reported infection and plasma inflammatory markers from early pregnancy showed two inflammatory markers were correlated with reported infection, but no correlation with any compounds in the metabolite profile was observed. We hypothesise that the lack of biomarkers of sPTB in the cervicovaginal fluid metabolome is simply because it lacks such markers in early pregnancy. We propose alternative biofluids be investigated for markers of sPTB. Our results lead us to call for greater scrutiny of previously published metabolomic data relating to biomarkers of sPTB in cervicovaginal fluids, as the use of small, high risk, or late pregnancy cohorts may identify metabolite biomarkers that are irrelevant for predicting risk in normal populations.

## 1. Introduction

Spontaneous preterm birth (sPTB) results from spontaneous onset of labour, or following preterm premature rupture of membrane (PPROM), resulting in birth before 37 weeks of gestation [1]. Preterm birth is a significant contributor to neonatal morbidity and mortality and is estimated to account for between 5%–18% of all live births worldwide [2]. Infants born before term are at increased risk of long term complications such as respiratory [3] and neurodegenerative disorders, learning disabilities [4,5], and early-onset diabetes [6]. An accurate screening test would enable healthcare providers to use existing preventative interventions (e.g., progestogens and cervical pessaries) and could accelerate the introduction of new interventions [7]. Currently, there are no available accurate tests able to predict sPTB at an early stage of gestation [8].

Previous investigations for biomarkers of preterm birth have focused almost exclusively on proteomic analysis and while this approach did not initially produced any reliable predictors of preterm birth [9,10,11], there have been promising advances recently [12]. Biomarker discovery for other complications of pregnancy has shown significant progress through metabolomic profiling of other body fluids such as urine [13]. Metabolomic technologies [14] focus on low molecular weight (<1 kDa) molecules which are the products of cellular and environmental interaction (metabolites). Depending on the biosample used, metabolite profiling provides a “snapshot” in time, of the functional phenotype [15]. The development of high-throughput mass spectral data acquisition technologies for metabolomic analysis has facilitated major progress in biomarker discovery, quantification and validation when coupled with access to large cohort sample biobanks [9].

### Study Design

The objective of our study was to look for early pregnancy metabolite biomarkers for sPTB. The use of cervicovaginal fluids in our experimental design was based on the hypothesis that a subclinical inflammatory process in the cervix is the final common pathway that leads to sPTB [16].

All women in our study were assessed as “low-risk” at the start of pregnancy. A low-risk sPTB population was selected in order to test the hypothesis that early universal biomarkers of sPTB would be detectable in a population with no known early indicators of sPTB. Previous studies had used either very small cohorts, high-risk cohorts, or took samples from a late gestational period [17,18] and are thus of dubious value for reliable prediction of sPTB.

The clinical characteristics of the participants for our study are detailed in Table 1. There were no statistically significant differences between the two groups in age, body mass index (BMI was determined at the antenatal booking visit) or ethnicity.

**Table 1:** Demographic and clinical characteristics of the cervicovaginal fluid study population. The cervicovaginal samples were collected at 20 weeks gestation.

Characteristic	sPTB Cases (*n* = 30)	Controls (*n* = 30)
Maternal Age (years) ^a^	32 ± 3.2	32 ± 3.5
Maternal BMI (Body Mass Index) ^a^	24.9 ± 4.5	24.7 ± 3.5
Ethnicity	27 Caucasians, 3 Asians	27 Caucasians, 3 Asians
Gestational age at the delivery (weeks)	34.3 ± 2.0 *	39.9 ± 0.9 *
Gestational age at sampling (weeks)	20.1 ± 0.7	19.6 ± 0.8
Cigarette smokers ^a^	29/30 did not smoke at recruitment	None smoked at recruitment
Shortest transvaginal cervical length at 20 weeks gestation (mm)	40.3 ± 8.9	39.5 ± 5.7
PPROM	11/30	-
Previous termination at >10 weeks’	1/30	-
Vaginal bleeding during pregnancy ^b^	8/30	4/30
Proven vaginal candida infection in pregnancy ^b^	2/30	2/30
Any infection during pregnancy (gasteroentiritis, pyelonephritis, vaginal candida ^b^	17/30	12/30
Fertility treatment to conceive current pregnancy	7/30	1/30

* *p* < 0.001; ^a^ Data from the time of recruitment; ^b^ Before 15 weeks’ SCOPE visit.

## 2. Results and Discussion

### 2.1. Metabolites Present in the Cervicovaginal Fluid

A total of 112 compounds were extracted from cervicovaginal fluid using GC-MS analysis. Relative abundances and variation for all compounds are given in Figure S1. (Supplementary materials). The metabolites identified were predominantly amino acids, followed by organic acids and fatty acids. A significant number of features were not able to be identified using our mass spectral libraries (“unknowns”). Four samples were identified as spurious and were excluded from the analysis.

The Student *T*-test [19] was used to look for differences between the sPTB cases and controls. After data normalisation and correction for laboratory contamination and batch effects, three weakly positive correlations (*p*~0.03–0.04) were evident (*T*-test *p*-values, Supplementary Table S1). However, calculation of the false discovery rate (*q*) showed that, even with the most generous test parameters [20,21], our metabolite data produced high *q*-values (0.99), casting doubt on the correlations between sPTB cases and these compounds (*T*-test *q*-values, Supplementary Table S1).

### 2.2. Inflammatory Markers, Infection and Metabolite Profile

We then considered the hypothesis that metabolite differences might be seen only in a subgroup of cases defined by infection. Infection is commonly reported as a major cause of spontaneous preterm delivery [22]. The interplay between infection and inflammation can trigger the release of factors that can cause cervical transformation and initiate uterine contractions [23]. Approximately half of the participants in our study had a history of infections during pregnancy (pyelonephritis, gastroenteritis and vaginal candida). Of the 30 sPTB cases, 17 reported a non-specific infection, and 12 of the 30 controls reported a non-specific infection. The data was divided into four groups (infected controls, non-infected controls, infected cases and non-infected cases) and a Kruskal Wallis test [24] was used to test for differences between these four groups. However the one weakly significant compound (*p* = 0.03) had a false discovery rate of 0.4, which made a true correlation unlikely (KW *q*-values, Supplementary Table S1).

These results led us to check the association between reported infection and known inflammatory markers. Inflammatory markers from plasma collected by another SCOPE research group (L.C.K.) were measured at 15 weeks gestation, and infection data was collected at 20 weeks gestation. Six inflammatory markers (CRP 120, MMP-9 51a, TIMP-1 126, IL-1ra 31a, CXCL10 16a, TNFR1A 80b) were selected based on the literature and compared with infection and case control status (Table 2). Two-way ANOVA without interactions was used, as initial tests showed that interactions were not significant, and all of the ANOVA assumptions were satisfied. Two markers for inflammation (CXCL10 16a and IL-1ra31a) were identified as strongly correlating to infection status (both *p* < 0.01), with supporting *q*-values of 0.03 and 0.05, respectively (Table 2). There was no correlation between the six selected inflammatory markers and sPTB status (Table 2).

**Table 2:** *p*-Values of infection and case/ctrl status when predicting log(inflammatory marker) in an additive case control model.

Marker	Infection (*p*)	Infection (*q* *)	sPTB-control (*p*)	sPTB-control (*q*)
CXCL10 16a	0.002	0.029	0.889	0.889
IL-1ra31a	0.008	0.046	0.181	0.363
TNFR1A 80b	0.079	0.236	0.669	0.803
MMP9 51a	0.151	0.362	0.530	0.794
TIMP-1 126	0.278	0.477	0.782	0.853
CRP 120	0.644	0.803	0.057	0.229

* Benjamini and Hochberg *q*-value.

Inflammatory marker data was compared with the cervicovaginal swab metabolite profiles. Tests of correlation between the log metabolite measurement and log inflammatory proxy estimates gave 17 *p*-values <0.05; of these, two *p*-values were <0.01 (Supplementary Table S2). Eleven of the 17 values were for metabolites correlated with CRP120. However, both false discovery rate calculations (Tibshirani and Storey, and Benjamini and Hochberg) gave *q*-values >0.89 for all tests, demonstrating that the statistical correlations could be due to random chance.

### 2.3. Discussion

Metabolomic analysis of cervicovaginal fluids was performed to determine whether metabolic alterations in early pregnancy were associated with the occurrence of sPTB. No significant change in the metabolite profile was observed between sPTB cases and normal term controls. While two inflammatory markers showed a correlation with reported infection, there was no correlation between reported infection and metabolites from the cervicovaginal fluids.

These results may be due to several reasons. The extraction and analytical method we used was optimised for the detection of amino acids, organic acids and fatty acids and we do not dispute that additional analysis using an alternative derivatisation method, LC-MS or NMR would have been desirable. Had we been able to obtain the entire cervicovaginal swab, we would have undertaken additional analyses, but only a single 250 µL aliquot of sample was available from the SCOPE biobank. Despite this, our chosen GC-MS methylchlorofomate derivatisation method produces derivatives that are more stable than other more common derivatisation techniques [25] and has been proven to be robust and sensitive to the metabolic changes associated with early gestational diabetes mellitus [26], so we were confident that if metabolic changes were occurring, we would have detected them. However, we believe that the type of biosample has a significant impact on likelihood of finding biomarkers. Alternative biofluids such as urine have shown potential for biomarkers of sPTB [13] and may be a better option for future studies.

A previous analysis in 2011 by Auray-Blais *et al.*, used liquid chromatography-mass spectrometry (LC-MS) to identify biomarkers of sPTB in cervicovaginal fluids [27]. However, the Auray-Blais study sampled only 15 women and samples were collected between 31–33 weeks of gestation. Given the lateness of sampling in the gestational period, any changes in the metabolome would be obvious, however the utility of biomarkers from that study as early predictors of sPTB has yet to be demonstrated. Perhaps more concerning is that the samples had been mixed with bovine serum albumin (BSA). As part of the method development for our study, we tested samples with BSA added and discovered that it contributed as many as eight extra compounds to the metabolite profile, which adds unnecessary complexity to the analysis. Furthermore, the PLSDA analysis used in the Auray-Blais study was not subjected to tests of out-of-sample prediction accuracy. Such an assessment is necessary to account for the large number of compounds screened, analogous to our use of *q*-values to estimate the likelihood of false discovery when large numbers of tests have been performed. Broadhurst *et al.* and others [28,29,30] have previously reported the need for statistical estimates of false discovery in metabolomics wherever *p*-values are used as measures of statistical significance, in order to avoid reporting false correlations.

A more recent study by Ghartey *et al.* [31], reported biomarkers from a sample of 20 women (10 sPTB, 10 term) sampled at 20–24 weeks and 24–28 weeks. However, unlike our study, this study specifically targeted women who were at a high risk of sPTB. We would question the utility of biomarkers identified from a high risk population as early predictors of sPTB risk in a population of unknown risk. Our study cohort was specifically chosen to be at low risk of sPTB, as the whole point of an early test for sPTB is for it to have predictive power in a population with unknown risk of sPTB.

## 3. Experimental Section

### 3.1. Sample Collection

Maternal cervicovaginal fluids were obtained at 20 weeks gestation from the New Zealand cohort of the Screening for Pregnancy Endpoints (SCOPE) biobank (http://www.scopestudy.net/). Informed consent was obtained from the patients recruited for the SCOPE study, and ethics approval was granted by the Auckland Ethics Committee (AKX/02/00/364). Cervicovaginal fluids were collected using a sterile cotton tipped swab and a sterile speculum. Sample swabs were collected into phosphate buffered saline solution (Na_2_HPO_4_·2H_2_O (3 mM); NaCl (404 mM); KH_2_PO_4_ (1 mM); sodium azide (0.001%)). A protease inhibitor cocktail (Roche, Basel, Switzerland) comprising phenylmethylsulfonyl fluoride (200 mM), *N*-[*N*-(l-3-*trans*-carboxyirane-2-carbonyl)-l-leucyl]-agmatine (10 mg/mL) and pepstatin (1 mg/mL) was added to arrest proteolytic enzyme activity during sample storage [32]. Samples were stored at −80 prior to extraction and analysis.

### 3.2. Sample Selection and Randomisation

Samples from sPTB cases were matched according to the maternal age and ethnicity with uncomplicated pregnancies. Samples were randomised prior to receipt by our laboratory, so that preparation and analysis was carried out blinded to clinical data. Samples were selected arbitrarily for preparation and analysis; however the ratio of approximately equal numbers of cases and controls was preserved in each analytical batch.

### 3.3. GC-MS Sample Preparation

Method development for GC-MS sample extraction and derivatisation was based on the protocol by Smart *et al.* [33]. Briefly, the cervicovaginal fluids were weighed into pre-weighed microcentrifuge tubes, (weighing error of ±0.29 mg) and 20 µL of 10 mM l-Alanine-2,3,3,4-d_4_ (Sigma-Aldrich, St. Louis, MO, USA) was added as an internal standard. Samples were stored on dry ice throughout the sample preparation process. Samples were dried for 4 h at 0.8 HPa in a centrifugal vacuum concentrator with a −104 °C refrigerated vapour trap (Savant SP5121P, Speedvac, Thermofisher, Waltham, MA, USA). Metabolites were extracted from the dried sample by addition of a 50% cold methanol-water solution (*v/v*: Merck KGaA, Darmstadt, Germany), vortexing, centrifugation (3000× *g* for 5 min at 4 °C), collection of the extract then re-extraction of the pellet by the addition of an 80% cold methanol-water solution. After vortexing and centrifugation, the supernatants from both the extractions were combined and dried in a centrifugal vacuum concentrator using the conditions specified above. The samples were then derivatized with methyl chloroformate prior to analysis.

### 3.4. GC-MS Data Acquisition

Gas Chromatography-Mass Spectrometry (GC-MS) was used for identification of amino acids, organic acids, and fatty acids. GC-MS instrument parameters were based on Smart *et al.* [33]. The instrument used was a7890A gas chromatograph coupled to an 5975C inert single quadrupole mass spectrometer (Agilent, Santa Clara, CA, USA). One microliter of sample was injected using a CTC PAL autosampler into a glass split/splitless 4 mm ID straight inlet liner packed with deactivated glass wool. The inlet was set to 290 °C, splitless, with a column flow 1.0 mL/min, with a column head pressure of 9 psi. Purge flow was set to 25 mL/min 1 min after injection. The column was a fused silica Zebron ZB-1701 30 m long, 0.25 mm ID, with a 0.15 µm stationary phase of 86% dimethylpolysiloxane and 14% cyanopropylphenyl (Phenomenex, Torrance, CA, USA). Carrier gas was instrument grade helium (99.99%, BOC). GC oven temperature programming started isothermally at 45 °C for 2 min, increased 9 °C/min to 180 °C, held 5 min; increased 40 °C/min to 220 °C, held 5 min; increased 40 °C/min to 240 °C, held 11.5 min; increased 40 °C/min to 280 °C, and held 2 min. The transfer line to the mass spectrometric detector was maintained at 250 °C, the source at 230 °C and quadrupole at 150 °C. The detector was turned on 5.5 min into the run. The detector was run in positive ionisation mode, with an electron energy of 70 eV. Data was acquired in scan mode with a range from 38 to 550 atomic mass units, and a detection threshold of 100 ion counts. Mass spectrometer detector calibration was carried out prior to analysis, and the septum and liner was changed every 200 injections. One solvent blank was run for every 6 sample injections to check for instrument carryover. One negative control and one quality control sample were run each day.

### 3.5. GC-MS Data Extraction and Compound Identification

Data processing was automated. The raw files obtained from the GC-MS were converted into CDF format and deconvoluted using the Automated Mass Spectral Deconvolution and Identification System (AMDIS—http://www.amdis.net/). An in-house mass spectral library and the NIST05 library was used for identification of compounds. The in-house library contained mass spectra primarily obtained from reference standards. Retention time and mass spectrum were used to match the compounds against the in-house library. Mass spectrum alone was used to match spectra against the NIST library and matches between 70% and 80% without a retention time have been labelled as “tentative”. A low match factor (1%) was used to prevent exclusion bias against compounds that were not present in the mass spectral libraries. Compounds with a match factor <70% from either library were labelled as “unknown”. As the output from AMDIS often contains missing values, an R-script based on XCMS with a Windows graphic user interface developed by Ting-Li Han was used to process the data offline. Briefly, the R-script returns a value for the retention time bin specified by AMDIS for a particular compound across all samples. The values reported are the maximum height at the apex of the most intense ion for the compound peak. Data were checked against negative controls to identify analytical contaminants. Peaks not deconvoluted by AMDIS were manually integrated.

### 3.6. Statistical Analysis

Data was normalised to the internal standard. Biomass was also measured for cervicovaginal swabs but normalisation by biomass had little effect on the results. Statistical analysis of the mass spectral data was performed using R. Welch’s *T*-Test, which is robust to non-normality at moderate sample size, was carried out on log-transformed, normalised data to compare case and control groups. Zero values were treated as missing values. The Kruskal Wallis test was used to look at the simultaneous influence of infection and case-control status. The four groups defined by these two variables each contain fewer individuals, so this non parametric test was used to provide robustness from non-normality, and also allowed this analysis to be carried out on the original scale without elimination of zeroes. Results were given in terms of *p*-value, and the threshold chosen for significance testing was *p* < 0.05 (5%). The false discovery rate measures the balance between true positives and false positives and gives an estimate of how many significant features will be false discoveries [21]. For example, a *q*-value of 0.9 (90%) is more likely to be a false positive, whereas a *q*-value of 0.05 (5%) is more likely to be a true positive. Both the Benjamini-Hochberg [20] and Storey-Tibshirani [21] false discovery rate calculations were employed. However, the latter were only used when the number of tests exceeded 50. In general, the Storey and Tibshirani values are less conservative and are reported. We did not attempt to build classification tree models as our results were not significant enough to warrant further investigation.

## Supplementary Materials

Supplementary materials can be found at http://www.mdpi.com1422-0067/16/11/26052/s1.

## References

[1] Blencowe H., Cousens S., Chou D., Oestergaard M., Say L., Moller A.-B., Kinney M., Lawn J. (2013). Born too soon: The global epidemiology of 15 million preterm births. Reprod. Health.

[2] Blencowe H., Cousens S., Oestergaard M.Z., Chou D., Moller A.-B., Narwal R., Lawn J.E. (2012). National, regional, and worldwide estimates of preterm birth rates in the year 2010 with time trends since 1990 for selected countries: A systematic analysis and implications. Lancet.

[3] Ball G., Boardman J.P., Aljabar P., Pandit A., Arichi T., Merchant N., Rueckert D., Edwards A.D., Counsell S.J. (2013). The influence of preterm birth on the developing thalamocortical connectome. Cortex.

[4] Nosarti C., Nam K.W., Walshe M., Murray R.M., Cuddy M., Rifkin L., Allin M.P.G. (2014). Preterm birth and structural brain alterations in early adulthood. Neuroimage Clin..

[5] Crump C., Winkleby M.A., Sundquist K., Sundquist J. (2011). Risk of diabetes among young adults born preterm in Sweden. Diabetes Care.

[6] Auger N., Abrahamowicz M., Wynant W., Lo E. (2014). Gestational age-dependent risk factors for preterm birth: Associations with maternal education and age early in gestation. Eur. J. Obetet. Gynecol. Reprod. Biol..

[7] Iams J.D. (2014). Clinical Practice: Prevention of preterm parturition. N. Engl. J. Med..

[8] Menon R., Jones J., Gunst P.R., Kacerovsky M., Fortunato S.J., Saade G.R., Basraon S. (2014). Amniotic fluid metabolomic analysis in spontaneous preterm birth. Reprod. Sci..

[9] Conde-Agudelo A., Papageorghiou A.T., Kennedy S.H., Villar J. (2011). Novel biomarkers for the prediction of the spontaneous preterm birth phenotype: A systematic review and meta-analysis. Eur. J. Obetet. Gynecol. Reprod. Biol..

[10] Honest H., Bachmann L.M., Gupta J.K., Kleijnen J., Khan K.S. (2002). Accuracy of cervicovaginal fetal fibronectin test in predicting risk of spontaneous preterm birth: Systematic review. BMJ.

[11] Stout M.J., Goetzinger K.R., Tuuli M.G., Cahill A.G., Macones G.A., Odibo A.O. (2012). First trimester serum analytes, maternal characteristics and ultrasound markers to predict pregnancies at risk for preterm birth. Placenta.

[12] McDonald C.R., Darling A.M., Conroy A.L., Tran V., Cabrera A., Liles W.C., Wang M., Aboud S., Urassa W., Fawzi W.W. (2015). Inflammatory and angiogenic factors at mid-pregnancy are associated with spontaneous preterm birth in a cohort of Tanzanian Women. PLoS ONE.

[13] Maitre L., Fthenou E., Athersuch T., Coen M., Toledano M., Holmes E., Kogevinas M., Chatzi L., Keun H. (2014). Urinary metabolic profiles in early pregnancy are associated with preterm birth and fetal growth restriction in the Rhea mother-child cohort study. BMC Med..

[14] Inoue K., Tanada C., Sakamoto T., Tsutsui H., Akiba T., Min J.Z., Todoroki K., Yamano Y., Toyo Oka T. (2015). Metabolomics approach of infant formula for the evaluation of contamination and degradation using hydrophilic interaction liquid chromatography coupled with mass spectrometry. Food Chem..

[15] Power K.M., Sanchez-Galan J.E., Luskey G.W., Koski K. G., Burns D.H. (2011). Use of near-infrared spectroscopic analysis of second trimester amniotic fluid to assess preterm births. J. Pregnancy.

[16] Liong S., di Quinzio M.K.W., Fleming G., Permezel M., Rice G.E., Georgiou H.M. (2013). Prediction of spontaneous preterm labour in at-risk pregnant women. Reproduction.

[17] Alleman B.W., Smith A.R., Byers H.M., Bedell B., Ryckman K.K., Murray J.C., Borowski K.S. (2013). A proposed method to predict preterm birth using clinical data, standard maternal serum screening, and cholesterol. Am. J. Obstet. Gynecol..

[18] Heazell A.E.P., Bernatavicius G., Warrander L., Brown M.C., Dunn W.B. (2012). A metabolomic approach identifies differences in maternal serum in third trimester pregnancies that end in poor perinatal outcome. Reprod. Sci..

[19] Utts J.M., Heckard R.F. (2006). Statistical Ideas and Methods.

[20] Benjamini Y., Hochberg Y. (1995). Controlling the false discovery rate: A practical and powerful approach to multiple testing. J. R. Stat. Soc. Ser. B.

[21] Storey J.D., Tibshirani R. (2003). Statistical significance for genomewide studies. Proc. Natl. Acad. Sci. USA.

[22] Sorokin Y., Romero R., Mele L., Wapner R.J., Iams J.D., Dudley D.J., Spong C.Y., Peaceman A.M., Leveno K.J., Harper M. (2010). Maternal serum interleukin-6, C-reactive protein, and matrix metalloproteinase-9 concentrations as risk factors for preterm birth <32 weeks and adverse neonatal outcomes. Am. J. Perinatol..

[23] Combs C.A., Gravett M., Garite T.J., Hickok D.E., Lapidus J., Porreco R., Rael J., Grove T., Morgan T.K., Clewell W. (2014). Amniotic fluid infection, inflammation, and colonization in preterm labor with intact membranes. Am. J. Obstet. Gynecol..

[24] Hollander M., Wolfe D.A. (1973). Nonparametric Statistical Methods.

[25] Villas-Bôas S.G., Smart K.F., Sivakumaran S., Lane G.A. (2011). Alkylation or silylation for analysis of amino and non-amino organic acids by GC-MS?. Metabolites.

[26] De Seymour J.V., Conlon C.A., Sulek K., Villas Bôas S.G., McCowan L.M.E., Kenny L.C., Baker P.N. (2014). Early pregnancy metabolite profiling discovers a potential biomarker for the subsequent development of gestational diabetes mellitus. Acta Diabetol..

[27] Auray-Blais C., Raiche E., Gagnon R., Berthiaume M., Pasquier J.-C. (2011). Metabolomics and preterm birth: What biomarkers in cervicovaginal secretions are predictive of high-risk pregnant women?. Int. J. Mol. Sci..

[28] Kell D.B. (2007). Metabolomic biomarkers: search, discovery and validation. Expert Rev. Mol. Diagn..

[29] Isaaq H.L., Veenstra T.D. (2013). Analytical methods and biomarker validation. Proteomic and Metabolomic Approaches to Biomarker Discovery.

[30] Broadhurst D.I., Kell B.D. (2006). Statistical strategies for avoiding false discoveries in metabolomics and related experiments. Metabolomics.

[31] Ghartey J., Bastek J.A., Brown A.G., Anglim L., Elovitz M.A. (2015). Women with preterm birth have a distinct cervicovaginal metabolome. Am. J. Obstet. Gynecol..

[32] Ayache S., Panelli M., Marincola M.F., Stroncek D.F. (2006). Effects of storage time and exogenous protease inhibitors on plasma protein levels. Am. J. Clin. Pathol..

[33] Smart K.F., Aggio R.B.M., van Houtte J.R., Villas-Bôas S.G. (2010). Analytical platform for metabolome analysis of microbial cells using methyl chloroformate derivatization followed by gas chromatography-mass spectrometry. Nat. Protoc..

