# Supplementary material for: Metabolite Profile of Cervicovaginal Fluids from Early Pregnancy Is Not Predictive of Spontaneous Preterm Birth
Source: Int J Mol Sci. 2015 Nov 19;16(11):27741–8. doi: 10.3390/ijms161126052 (PMC4661910; doi:10.3390/ijms161126052)
Supplement: Supplementary file 1 [file ijms-16-26052-s001.pdf]

Supplementary Materials: Metabolite Profile of Cervicovaginal Fluids from Early Pregnancy is not Predictive of Spontaneous Preterm Birth

Melinda M. Thomas, Karolina Sulek, Elizabeth J. McKenzie, Beatrix Jones, Ting-Li Han, Silas G. Villas-Boas, Louise C. Kenny, Lesley M.E. McCowan and Philip N. Baker

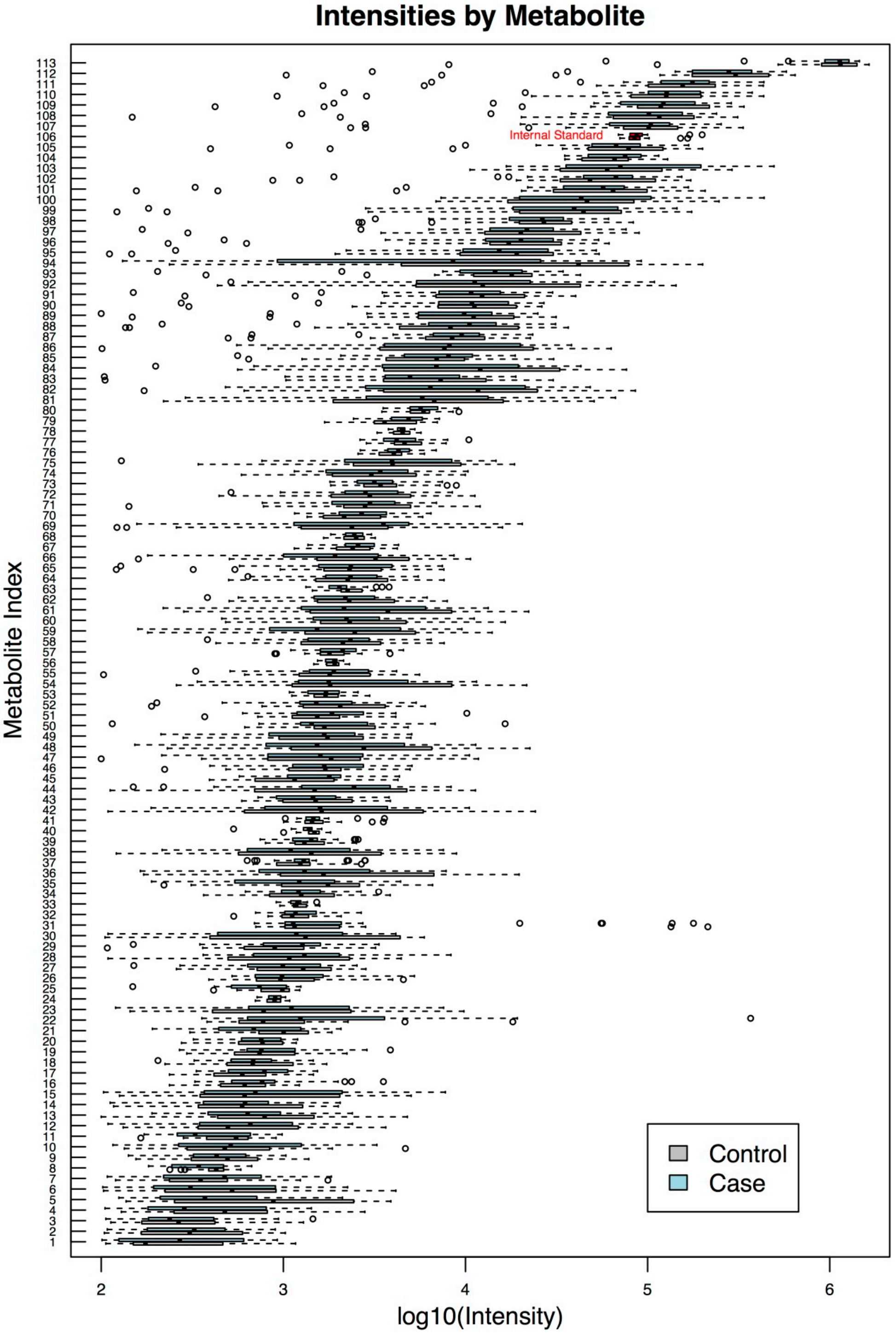

Figure S1. Relative abundances of metabolites from cervicovaginal swabs, showing the median and distribution for each metabolite. The internal standard is also shown (red font).

Index of compounds shown in Figure S1.

Index	Compound	Index	Compound
1	Unknown 38	57	Unknown 40
2	Unknown 20	58	4-Amino-5-methyl-2-hexenoic acid (tentative)
3	Phthalimide (tentative)	59	Unknown 44
4	Unknown 24	60	Unknown 43
5	N-tert-Butylcinnamamide (tentative)	61	Unknown 30
6	Unknown 23	62	Unknown 18
7	Unknown 13	63	Unknown 4
8	Unknown 5	64	Unknown 14
9	Unknown 37	65	Unknown 34
10	Unknown 19	66	Unknown 27
11	Unknown 3	67	Unknown 32
12	Unknown 28	68	Unknown 22
13	NADP/NADPH (tentative)	69	Benzenepropanoic acid, α-thiocyanato
14	Glyoxylic acid, (*p*-methoxyphenyl)	70	Unknown 1
15	4-Hydroxycinnamic acid (tentative)	71	Unknown 15
16	Unknown 16	72	Unknown 17
17	Capric acid	73	Benzoic acid
18	Benzaldehyde	74	2-Oxoleucine
19	2-Hydroxyglutaramic acid	75	Cysteine
20	Carbamic acid	76	Palmitic acid
21	Benzoic acid oxime	77	Unknown 46
22	Citric acid	78	10-Nonadecanamine (tentative)
23	Proline derivative 1	79	Lauric acid
24	Unknown 10	80	Stearic acid
25	Unknown 45	81	2-Oxoglutaric acid derivative 2 (tentative)
26	3-Aminoisobutyric acid	82	Leucine derivative 1
27	Succinic acid derivative 1	83	Serine
28	*trans*-Cinnamic acid	84	Glycine derivative 2 (tentative)
29	Unknown 39	85	Unknown 35
30	Proline derivative 3	86	Alanine derivative 2 (tentative)
31	Unknown 33	87	Unknown 31
32	Caprylic acid	88	2-Oxoglutamaric acid (tentative)
33	Unknown 12	89	Tryptophan
34	Unknown 7	90	Glutathione
35	Unknown 25	91	Creatinine
36	Unknown 21	92	Unknown 36
37	Undecane	93	Methionine
38	Lactic acid derivative 1	94	Ornithine
39	Unknown 42	95	Asparagine
40	Unknown 8	96	Pyroglutamic acid
41	Unknown 41	97	Threonine
42	Unknown 26	98	Unknown 9
43	Phenylhydrazine	99	Lysine
44	Histidine	100	Succinic acid derivative 2 (tentative)
45	Mesitylenic acid	101	Tyrosine
46	1,2,4-Triazole-3-thione (tentative)	102	Proline derivative 2
47	4-Methoxyphenylacetamide (tentative)	103	Butyric acid
48	Unknown 29	104	Phenol, 2,4-bis(1,1-dimethylethyl)
49	2-Oxoglutaric acid derivative 1	105	Phenylalanine
50	2-Aminobutyric acid	107	Glycine derivative 1
51	Oxalic acid	108	Glutamic acid
52	Isosafrole (tentative)	109	Aspartic acid
53	Unknown 2	110	Valine
54	2-Pyrrolidinone, 5-(cyclohexylmethyl)	111	Alanine derivative 1
55	2-Aminoadipic acid	112	Leucine/Isoleucine derivative
56	Unknown 11	113	Lactic acid derivative 2

Table S1. Left column: list of compounds identified from cervicovaginal fluids with *p*- and *q*-values (Student *T*-test and Storey-Tibshirani, respectively) for sPTB cases *vs.* controls. Right column: *p*-values from a Kruskal-Wallis test for differences across the four groups defined by sPTB and reported infection.

Compound	*T*-Test *p*-Value	*T*-Test *q*-Value	Compound	KW Test *p*-Value	KW Test *q*-Value
Benzoic acid oxime	0.029	0.999	Undecane	0.029	0.387
Unknown 5	0.029	0.999	Benzoic acid oxime	0.130	0.387
N-tert-Butylcinnamamide (tentative)	0.044	0.999	Unknown 12	0.135	0.387
Unknown 45	0.049	0.999	Mesitylenic acid	0.171	0.387
Mesitylenic acid	0.059	0.999	Citric acid	0.190	0.387
Unknown 16	0.069	0.999	Unknown 5	0.208	0.387
Unknown 8	0.072	0.999	Unknown 8	0.219	0.387
Ornithine	0.081	0.999	Unknown 42	0.265	0.387
Unknown 3	0.085	0.999	Glutamic acid	0.268	0.387
Citric acid	0.131	0.999	Unknown 4	0.271	0.387
Unknown 4	0.133	0.999	Unknown 11	0.307	0.387
Lauric acid	0.136	0.999	Unknown 27	0.315	0.387
Unknown 44	0.156	0.999	Phenylhydrazine	0.326	0.387
Unknown 27	0.171	0.999	10-Nonadecanamine (tentative)	0.332	0.387
Unknown 24	0.196	0.999	Unknown 23	0.332	0.387
Capric acid	0.234	0.999	Unknown 3	0.334	0.387
Isosafrole (tentative)	0.259	0.999	Unknown 20	0.337	0.387
Unknown 21	0.292	0.999	2-Oxoglutaric acid derivative 1	0.359	0.387
Unknown 39	0.295	0.999	Unknown 16	0.362	0.387
Unknown 25	0.295	0.999	Unknown 25	0.378	0.387
Unknown 46	0.298	0.999	2-Oxoleucine	0.378	0.387
Unknown 23	0.300	0.999	Unknown 10	0.397	0.387
Butyric acid	0.305	0.999	Leucine/Isoleucine derivative 2	0.399	0.387
Glycine derivative 2 (tentative)	0.305	0.999	Pyroglutamic acid	0.399	0.387
Unknown 18	0.305	0.999	Lactic acid derivative 2	0.403	0.387
Unknown 41	0.322	0.999	NADP/NADPH (tentative)	0.408	0.387
Unknown 12	0.324	0.999	Tryptophan	0.415	0.387
Oxalic acid	0.330	0.999	Proline derivative 2	0.416	0.387
Unknown 43	0.331	0.999	Unknown 28	0.427	0.387
Serine	0.338	0.999	2-Hydroxyglutaramic acid	0.434	0.387
Benzenepropanoic acid, α-thiocyanato	0.339	0.999	Unknown 45	0.436	0.387
Unknown 29	0.341	0.999	Unknown 46	0.441	0.387
Unknown 11	0.357	0.999	Benzaldehyde	0.444	0.387
Methionine	0.399	0.999	Creatinine	0.446	0.387
Succinic acid derivative 1	0.420	0.999	Ornithine	0.447	0.387
Unknown 30	0.423	0.999	Unknown 36	0.450	0.387
Unknown 40	0.426	0.999	Glyoxylic acid, (*p*-methoxyphenyl)	0.454	0.387
Unknown 6	0.440	0.999	2-Oxoglutamaric acid (tentative)	0.461	0.387
trans-Cinnamic acid	0.455	0.999	Unknown 37	0.461	0.387
Benzoic acid	0.455	0.999	Methionine	0.466	0.387

Table S1. *Cont.*

Compound	*T*-Test *p*-Value	*T*-Test *q*-Value	Compound	KW Test *p*-Value	KW Test *q*-Value
NADP/NADPH (tentative)	0.458	0.999	3-Aminoisobutyric acid	0.468	0.387
Proline derivative 1	0.474	0.999	Lauric acid	0.471	0.387
Unknown 32	0.497	0.999	Unknown 24	0.472	0.387
2-Aminobutyric acid	0.501	0.999	Isosafrole (tentative)	0.473	0.387
Tryptophan	0.510	0.999	Leucine derivative 1	0.478	0.387
Unknown 37	0.523	0.999	2-Pyrrolidinone, 5-(cyclohexylmethyl)	0.491	0.387
Unknown 2	0.523	0.999	Unknown 29	0.491	0.387
Creatinine	0.523	0.999	*trans*-Cinnamic acid	0.495	0.387
Unknown 13	0.530	0.999	Unknown 1	0.499	0.387
Alanine derivative 1	0.533	0.999	Unknown 26	0.515	0.387
Histidine	0.542	0.999	Unknown 35	0.517	0.387
Leucine derivative 1	0.542	0.999	Stearic acid	0.517	0.387
Unknown 26	0.547	0.999	Histidine	0.520	0.387
2-Pyrrolidinone, 5-(cyclohexylmethyl)	0.553	0.999	Lactic acid derivative 1	0.520	0.387
Unknown 36	0.562	0.999	Tyrosine	0.524	0.387
1,2,4-Triazole-3-thione (tentative)	0.573	0.999	Unknown 21	0.524	0.387
Proline derivative 3	0.578	0.999	Glutathione	0.527	0.387
2-Oxoglutamaric acid (tentative)	0.583	0.999	Lysine	0.537	0.387
Unknown 15	0.592	0.999	Unknown 41	0.538	0.387
10-Nonadecanamine (tentative)	0.600	0.999	Phenylalanine	0.540	0.387
Threonine	0.611	0.999	Caprylic acid	0.557	0.387
Glutathione	0.612	0.999	Unknown 44	0.559	0.387
Unknown 9	0.617	0.999	*N*-tert-Butylcinnamamide (tentative)	0.565	0.387
Phenol, 2,4-bis(1,1-dimethylethyl)	0.627	0.999	Unknown 2	0.568	0.387
2-Hydroxyglutaramic acid	0.629	0.999	Alanine derivative 2 (tentative)	0.584	0.387
Cysteine	0.629	0.999	Glycine derivative 2 (tentative)	0.590	0.387
Unknown 31	0.635	0.999	Unknown 15	0.599	0.387
Unknown 17	0.648	0.999	Palmitic acid	0.603	0.387
Lactic acid derivative 1	0.650	0.999	Phthalimide (tentative)	0.605	0.387
Unknown 22	0.674	0.999	Phenol, 2,4-bis(1,1-dimethylethyl)	0.617	0.387
Unknown 14	0.679	0.999	Unknown 38	0.626	0.387
Unknown 10	0.700	0.999	Benzoic acid	0.628	0.387
Caprylic acid	0.711	0.999	Unknown 6	0.629	0.387
Unknown 1	0.715	0.999	Unknown 39	0.633	0.387
2-Oxoglutaric acid derivative 1	0.718	0.999	Unknown 13	0.634	0.387
Valine	0.719	0.999	Unknown 22	0.638	0.387
Palmitic acid	0.733	0.999	Unknown 30	0.654	0.387
Benzaldehyde	0.744	0.999	Capric acid	0.658	0.387
Proline derivative 2	0.758	0.999	Unknown 9	0.661	0.387

Table S1. *Cont.*

Compound	*T*-Test *p*-Value	*T*-Test *q*-Value	Compound	KW Test *p*-Value	KW Test *q*-Value
Unknown 19	0.774	0.999	Glycine derivative 1	0.676	0.387
Glyoxylic acid, (*p*-methoxyphenyl)	0.775	0.999	Butyric acid	0.676	0.387
Unknown 35	0.779	0.999	Proline derivative 1	0.679	0.387
Asparagine	0.784	0.999	Unknown 18	0.692	0.387
Glycine derivative 1	0.795	0.999	Unknown 43	0.694	0.387
4-Amino-5-methyl-2-hexenoic acid (tentative)	0.802	0.999	2-Aminobutyric acid	0.694	0.387
2-Oxoglutaric acid derivative 2 (tentative)	0.817	0.999	Serine	0.698	0.387
Unknown 34	0.817	0.999	Benzenepropanoic acid, α-thiocyanato	0.700	0.387
Stearic acid	0.836	0.999	Aspartic acid	0.700	0.387
Unknown 42	0.837	0.999	1,2,4-Triazole-3-thione (tentative)	0.725	0.387
Phenylhydrazine	0.838	0.999	Valine	0.726	0.387
3-Aminoisobutyric acid	0.853	0.999	Unknown 17	0.727	0.387
Unknown 20	0.859	0.999	4-Hydroxycinnamic acid (tentative)	0.727	0.387
Succinic acid derivative 2 (tentative)	0.865	0.999	Proline derivative 3	0.732	0.387
Unknown 7	0.891	0.999	Unknown 19	0.759	0.394
Unknown 28	0.894	0.999	Oxalic acid	0.760	0.394
Aspartic acid	0.898	0.999	Unknown 14	0.790	0.401
Unknown 38	0.920	0.999	Cysteine	0.810	0.401
Pyroglutamic acid	0.924	0.999	Unknown 32	0.822	0.401
Lactic acid derivative 2	0.929	0.999	Unknown 31	0.824	0.401
Lysine	0.945	0.999	Carbamic acid	0.827	0.401
Glutamic acid	0.948	0.999	Threonine	0.844	0.401
Phthalimide (tentative)	0.948	0.999	2-Aminoadipic acid	0.845	0.401
Tyrosine	0.956	0.999	Alanine derivative 1	0.846	0.401
Leucine/Isoleucine derivative 2	0.962	0.999	Succinic acid derivative 1	0.847	0.401
Carbamic acid	0.964	0.999	Unknown 40	0.880	0.413
4-Hydroxycinnamic acid (tentative)	0.972	0.999	Unknown 34	0.901	0.418
2-Aminoadipic acid	0.974	0.999	Asparagine	0.908	0.418
4-Methoxyphenylacetamide (tentative)	0.976	0.999	2-Oxoglutaric acid derivative 2 (tentative)	0.917	0.418
Alanine derivative 2 (tentative)	0.991	0.999	Unknown 7	0.927	0.418
Phenylalanine	0.993	0.999	4-Methoxyphenylacetami de (tentative)	0.934	0.418
2-Oxoleucine	0.995	0.999	Succinic acid derivative 2 (tentative)	0.955	0.423
Undecane	0.999	0.999	4-Amino-5-methyl-2-hexe noic acid (tentative)	0.980	0.430

Table S2. Results from tests of correlation between inflammatory marker abundance at 15 weeks gestation with metabolites from cervicovaginal fluids collected at 20 weeks gestation.

Compound	Inflammatory Marker (*p*-Value)					
	**CRP 120**	**CXCL 10 16a**	**IL-1ra 31a**	**MMP-9 51a**	**TIMP-1 126**	**TNFR1A 80b**
Unknown 6	0.384	0.035	0.182	0.768	0.873	0.761
Benzoic acid oxime	0.275	0.042	0.214	0.980	0.134	0.116
2,4-Bis(1,1-dimethylethyl)-phenol	0.676	0.066	0.496	0.695	0.853	0.188
Unknown 5	0.190	0.069	0.211	0.634	0.270	0.508
Succinic acid derivative 1	0.556	0.075	0.011	0.170	0.104	0.271
Unknown 16	0.840	0.082	0.186	0.550	0.376	0.435
Citric acid	0.840	0.082	0.267	0.475	0.547	0.619
Unknown 3	0.256	0.088	0.311	0.625	0.098	0.260
Unknown 45	0.276	0.100	0.411	0.945	0.856	0.209
trans-Cinnamic acid	0.461	0.119	0.120	0.125	0.635	0.028
Phthalimide (tentative)	0.135	0.123	0.204	0.362	0.656	0.721
Unknown 23	0.111	0.128	0.053	0.367	0.306	0.950
Lysine	0.923	0.140	0.334	0.540	0.288	0.362
Unknown 46	0.291	0.143	0.278	0.433	0.584	0.572
Unknown 38	0.705	0.149	0.833	0.250	0.878	0.998
Asparagine	0.598	0.164	0.364	0.522	0.104	0.356
Succinic acid derivative 2 (tentative)	0.810	0.175	0.045	0.144	0.064	0.429
2-Aminoadipic acid	0.264	0.228	0.224	0.348	0.195	0.615
Carbamic acid	0.318	0.240	0.555	0.995	0.586	0.654
Aspartic acid	0.860	0.257	0.492	0.465	0.186	0.626
Valine	0.698	0.269	0.565	0.536	0.165	0.669
Unknown 27	0.058	0.271	0.052	0.938	0.307	0.521
Unknown 28	0.527	0.271	0.419	0.955	0.249	0.897
Unknown 11	0.100	0.278	0.795	0.830	0.098	0.880
Unknown 36	0.341	0.286	0.152	0.445	0.420	0.858
4-Hydroxycinnamic acid (tentative)	0.067	0.291	0.477	0.675	0.596	0.220
Unknown 29	0.226	0.295	0.094	0.642	0.150	0.669
Mesitylenic acid	0.512	0.303	0.320	0.735	0.264	0.075
Benzoic acid	0.845	0.304	0.419	0.163	0.054	0.038
Unknown 25	0.248	0.304	0.113	0.504	0.119	0.816
Unknown 21	0.148	0.305	0.105	0.759	0.132	0.838
Alanine derivative 1	0.710	0.306	0.797	0.743	0.149	0.796
Histidine	0.143	0.306	0.217	0.688	0.790	0.734
Tyrosine	0.840	0.308	0.522	0.363	0.302	0.581
Phenylalanine	0.853	0.315	0.501	0.379	0.240	0.650
Unknown 9	0.465	0.320	0.395	0.804	0.280	0.955
Oxalic acid	0.858	0.327	0.610	0.740	0.444	0.903
Proline derivative 3	0.819	0.327	0.783	0.963	0.123	0.893
Unknown 43	0.046	0.328	0.342	0.670	0.199	0.818
Glycine derivative 1	0.881	0.336	0.505	0.584	0.274	0.909
Unknown 30	0.215	0.341	0.099	0.518	0.142	0.586
Unknown 40	0.360	0.345	0.713	0.985	0.822	0.296
Unknown 4	0.146	0.350	0.290	0.797	0.082	0.664
Unknown 24	0.887	0.353	0.442	0.685	0.822	0.620
Unknown 31	0.949	0.353	0.757	0.909	0.168	0.916
Proline derivative 2	0.290	0.361	0.455	0.247	0.176	0.528
2-Oxoglutaric acid derivative 2 (tentative)	0.948	0.382	0.299	0.569	0.128	0.418
Isosafrole (tentative)	0.045	0.383	0.126	0.912	0.439	0.948

Table S2. *Cont.*

Compound	Inflammatory Marker (*p*-Value)					
	**CRP**	**CXCL 10**	**IL-1ra**	**MMP-9**	**TIMP-1**	**TNFR1A**
	**120**	**16a**	**31a**	**51a**	**126**	**80b**
Leucine/Isoleucine derivative 2	0.812	0.392	0.568	0.315	0.330	0.564
Unknown 14	0.379	0.402	0.492	0.680	0.365	0.599
Leucine derivative 1	0.166	0.406	0.191	0.867	0.341	0.985
Alanine derivative 2 (tentative)	0.207	0.411	0.309	0.736	0.307	0.750
Lactic acid derivative 2	0.871	0.419	0.708	0.210	0.520	0.841
3-Aminoisobutyric acid	0.226	0.432	0.525	0.692	0.613	0.204
Cysteine	0.028	0.434	0.190	0.842	0.472	0.789
Unknown 13	0.217	0.436	0.883	0.217	0.137	0.277
2-Oxoglutamaric acid (tentative)	0.900	0.439	0.575	0.565	0.216	0.990
Unknown 39	0.968	0.449	0.403	0.970	0.129	0.586
Serine	0.710	0.461	0.652	0.810	0.707	0.960
Lactic acid derivative 1	0.190	0.469	0.220	0.502	0.443	0.790
Unknown 18	0.024	0.479	0.342	0.439	0.282	0.498
Unknown 7	0.037	0.479	0.682	0.328	0.386	0.559
2-Hydroxyglutaramic acid	0.173	0.491	0.331	0.475	0.770	0.114
Unknown 34	0.826	0.498	0.913	0.411	0.348	0.769
Glutamic acid	0.798	0.508	0.738	0.327	0.210	0.744
Unknown 17	0.044	0.515	0.272	0.516	0.181	0.694
Palmitic acid	0.514	0.518	0.701	0.675	0.224	0.410
Unknown 8	0.033	0.520	0.711	0.709	0.829	0.924
Stearic acid	0.475	0.524	0.567	0.520	0.370	0.486
Unknown 15	0.233	0.527	0.418	0.399	0.440	0.407
Glycine derivative 2 (tentative)	0.027	0.528	0.136	0.896	0.453	0.617
Proline derivative 1	0.034	0.542	0.691	0.218	0.719	0.114
Threonine	0.892	0.560	0.652	0.989	0.364	0.848
Unknown 35	0.783	0.565	0.809	0.819	0.855	0.473
N-tert-Butylcinnamamide (tentative)	0.150	0.576	0.474	0.451	0.751	0.649
Unknown 41	0.493	0.583	0.905	0.637	0.643	0.460
4-Methyl-1,2,4-triazolethione (tentative)	0.128	0.589	0.752	0.768	0.169	0.609
NADP/NADPH (tentative)	0.193	0.592	0.287	0.788	0.125	0.677
Tryptophan	0.869	0.602	0.398	0.885	0.561	0.629
Methionine	0.977	0.604	0.375	0.575	0.595	0.735
Glutathione	0.655	0.626	0.887	0.521	0.365	0.902
10-Nonadecanamine (tentative)	0.106	0.631	0.873	0.784	0.134	0.549
Unknown 37	0.622	0.632	0.295	0.747	0.833	0.912
Benzenepropanoic acid, α-thiocyanato	0.844	0.661	0.752	0.258	0.070	0.689
Undecane	0.181	0.664	0.911	0.433	0.611	0.324
Unknown 19	0.505	0.703	0.379	0.200	0.139	0.527
Phenylhydrazine	0.544	0.713	0.377	0.527	0.922	0.981
Unknown 1	0.334	0.714	0.585	0.933	0.728	0.426
4-Methoxybenzeneacetamide (tentative)	0.129	0.723	0.665	0.728	0.161	0.563
Pyroglutamic acid	0.945	0.725	0.981	0.402	0.168	0.840
Unknown 26	0.104	0.726	0.396	0.947	0.213	0.797
Unknown 10	0.067	0.745	0.727	0.941	0.093	0.125
Butyric acid	0.012	0.748	0.199	0.595	0.825	0.500
Benzaldehyde	0.634	0.751	0.640	0.387	0.976	0.841

Table S2. *Cont.*

Compound	Inflammatory Marker (*p*-Value)					
	**CRP 120**	**CXCL 10 16a**	**IL-1ra 31a**	**MMP-9 51a**	**TIMP-1 126**	**TNFR1A 80b**
2-Pyrrolidinone, 5-(cyclohexylmethyl)	0.105	0.751	0.499	0.863	0.179	0.871
Unknown 2	0.292	0.753	0.777	0.927	0.096	0.972
Unknown 44	0.080	0.770	0.157	0.419	0.465	0.913
Unknown 20	0.501	0.821	0.389	0.440	0.728	0.587
Creatinine	0.819	0.835	0.538	0.725	0.534	0.833
Caprylic acid	0.347	0.847	0.468	0.818	0.211	0.977
4-Amino-5-methyl-2-hexenoic acid (tentative)	0.022	0.855	0.528	0.707	0.175	0.473
Unknown 12	0.270	0.863	0.908	0.818	0.093	0.434
Unknown 32	0.281	0.864	0.888	0.951	0.395	0.127
Capric acid	0.797	0.866	0.413	0.796	0.217	0.589
2-Oxoglutaric acid derivative 1	0.102	0.879	0.198	0.688	0.599	0.881
Unknown 42	0.726	0.891	0.749	0.387	0.583	0.917
2-Aminobutyric acid	0.425	0.912	0.985	0.546	0.965	0.973
2-Oxoleucine	0.583	0.963	0.599	0.383	0.412	0.993
Unknown 22	0.248	0.981	0.913	0.811	0.700	0.195
Lauric acid	0.623	0.982	0.411	0.985	0.157	0.671
Glyoxylic acid, (*p*-methoxyphenyl)	0.144	0.996	0.490	0.643	0.829	0.777
Ornithine	0.161	0.998	0.758	0.706	0.838	0.662
